# A Novel Sulfonamide, 4-FS, Reduces Ethanol Drinking and Physical Withdrawal Associated With Ethanol Dependence

**DOI:** 10.3390/ijms21124411

**Published:** 2020-06-21

**Authors:** Muhammad Sona Khan, Wulfran Trenet, Nancy Xing, Britta Sibley, Muzaffar Abbas, Mariya al-Rashida, Khalid Rauf, Chitra D. Mandyam

**Affiliations:** 1 Abbottabad Campus, COMSATS University Islamabad, Abbottabad, Khyber Pakhtunkhawa 22060, Pakistan; muhammadsona1986@gmail.com; 2 VA San Diego Healthcare System, San Diego, CA 92161, USA; wtrenet@vapop.ucsd.edu (W.T.); nxing@ucsd.edu (N.X.); bsibley@ucsd.edu (B.S.); 3 Department of Anesthesiology, University of California San Diego, San Diego, CA 92161, USA; 4 Department of Pharmacy, Capital University of Science & Technology, Islamabad 44000, Pakistan; muzaffar.abbas@cust.edu.pk; 5 Department of Chemistry, Forman Christian College, A Chartered University, Ferozepur Road, Lahore 54600, Pakistan; mariyarashida@fc.college.edu

**Keywords:** ethanol self-administration, carbonic anhydrase, hippocampus, GABA, GluR, Fos

## Abstract

Carbonic anhydrase (CA) is abundant in glial cells in the brain and CA type II isoform (CA II) activity in the hippocampus plays an important role in buffering extracellular pH transients produced by neural activity. Chronic ethanol exposure results in respiratory and metabolic acidosis, producing shifts in extracellular pH in the brain and body. These neurophysiological changes by ethanol are hypothesized to contribute to the continued drinking behavior and physical withdrawal behavior in subjects consuming ethanol chronically. We explored whether chronic ethanol self-administration (ethanol drinking, 10% *v*/*v*; ED) without or under the influence of chronic intermittent ethanol vapor (CIE-ED) experience alters the expression of CA II in the hippocampus. Postmortem hippocampal tissue analyses demonstrated that CA II levels were enhanced in the hilus region of the hippocampus in ED and CIE-ED rats. We used a novel molecule—4-fluoro-N-(4-sulfamoylphenyl) benzenesulfonamide (4-FS)—a selective CA II inhibitor, to determine whether CA II plays a role in ethanol self-administration in ED and CIE-ED rats and physical withdrawal behavior in CIE-ED rats. 4-FS (20 mg/kg, i.p.) reduced ethanol self-administration in ED rats and physical withdrawal behavior in CIE-ED rats. Postmortem hippocampal tissue analyses demonstrated that 4-FS reduced CA II expression in ED and CIE-ED rats to control levels. In parallel, 4-FS enhanced GABA_A_ receptor expression, reduced ratio of glutamatergic GluN2A/2B receptors and enhanced the expression of Fos, a marker of neuronal activation in the ventral hippocampus in ED rats. These findings suggest that 4-FS enhanced GABAergic transmission and increased activity of neurons of inhibitory phenotypes. Taken together, these findings support the role of CA II in assisting with negative affective behaviors associated with moderate to severe alcohol use disorders (AUD) and that CA II inhibitors are a potential therapeutic target to reduce continued drinking and somatic withdrawal symptoms associated with moderate to severe AUD.

## 1. Introduction

Alcohol use disorder (AUD) affects a significant population in the United States [1], and is associated with a plethora of neurological deficits. Particularly notable from clinical findings are the deficits dependent on the hippocampus, including, but not limited to, loss of brain volume and cognitive impairments [2,3,4,5,6]. Even more interesting is the fact that these impairments have been replicated in several widely accepted rodent models of alcohol dependence [7,8,9,10]. The chronic intermittent ethanol vapor exposure (CIE) model, is a widely used model that implements daily cycles of intoxication via ethanol vapors and withdrawal to induce clinical signs of alcoholism, such as somatic withdrawal symptoms and escalated ethanol drinking in rats [11,12]. Neurobiological alterations in the hippocampus produced by CIE could be associated with unregulated drinking patterns observed in CIE animals [13,14,15,16,17,18,19,20,21]. 

In the context of the above hypothesis, ethanol disrupts the delicate balance between γ-aminobutyric acid (GABA) and glutamate in the hippocampus. These neuroadaptations induced by ethanol could lead to behavioral deficits, including unregulated patterns of drinking and physical withdrawal behaviors [19]. For example, in vitro studies have demonstrated excitotoxicity in the hippocampus after withdrawal from chronic ethanol exposure and not during ethanol exposure [22,23,24]. Mechanistic studies using in vitro models have further shown that excessive release of glutamate and polyamines and corresponding activation of N-methyl-D-aspartate type glutamatergic receptors (GluNs) contribute to the excitotoxicity [25]. Supporting the in vitro studies, in vivo studies also show neurotoxicity in the hippocampus [26,27,28,29,30]. For example, during ethanol withdrawal, glutamate release is increased in the hippocampus [26,27], and the changes in glutamate levels are also associated with increased number of functional GluNs [31,32], suggesting that these effects may induce ethanol withdrawal hyperexcitability and may also lead to increased susceptibility to somatic withdrawal symptoms [33].

The neuroadaptations by ethanol also could produce neurophysiological effects via GABA/glutamate imbalance, including changes in extracellular pH, and alkaline transients in the brain, especially in the hippocampus [34,35]. Particularly interesting is the presence of extracellular carbonic anhydrases (CA) in the hippocampus, enzymes that regulate pH transients associated with glutamatergic and GABAergic transmission [34]. Notably, inhibition of CA activity is anticonvulsive [36,37]. For example, in humans, the CA inhibitor acetazolamide is used for adjuvant antiepileptic therapy [38,39], and in animals reduces the convulsant effects in a kindling model of partial epilepsy [40]. Furthermore, topiramate, a drug used to treat seizures, psychiatric and neurological conditions is a CA inhibitor, and has shown beneficial effects in reducing craving and drinking obsessions in subjects with AUD [41]. Given that chronic ethanol experience results in epileptiform activity in the hippocampus [42], we tested the hypothesis that systemic administration of a novel CA inhibitor 4-fluoro-N-(4-sulfamoylphenyl)benzenesulfonamide (4-FS; CA II inhibitor [43]) will reduce physical withdrawal and unregulated drinking associated with alcohol dependence in CIE rats. We also tested the subhypothesis that the reduced behaviors by 4-FS will be associated with reduced expression of CA II and enhanced expression of GABA receptors in the hippocampus. 

## 2. Results

### 2.1. CIE-ED Rats Consume More Ethanol Than ED Rats 

**Figure 1:**
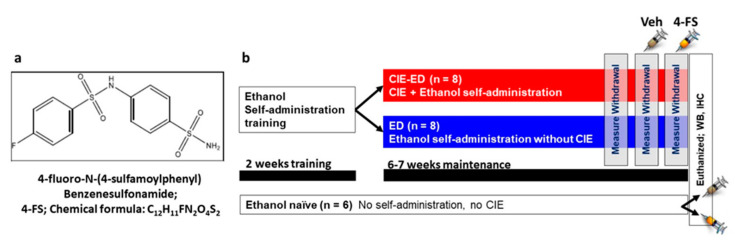
(**a**) Chemical structure of 4-FS. (**b**) Schematic of the experimental timeline and experimental group information.

**Figure 2:**
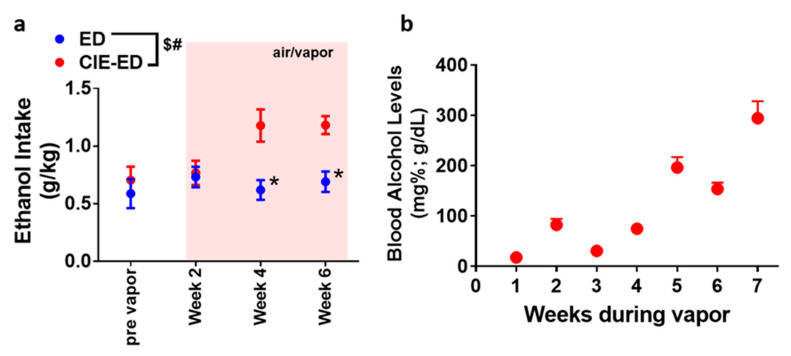
Ethanol self-administration and blood alcohol levels. (**a**) Amount of ethanol consumed prior to chronic intermittent ethanol (CIE) initiation and over the 6 weeks period of CIE or air exposure. Weeks of air/vapor exposure are indicated over a pale pink background. (**b**) Blood alcohol levels measured once a week in animals experiencing CIE. CIE-ED, *n* = 7 and ED, *n* = 8. $, interaction; #, main effect of groups; * *p* < 0.05 vs. CIE-ED by two-way analysis of variance (ANOVA). Data are expressed as mean ± S.E.M.

Experimental design, timeline of vapor exposure and treatment groups are indicated in Figure 1. Ethanol (10% *v*/*v*) consumption was determined in CIE-ED and ED rats prior to the onset of vapor exposure and during vapor exposure weeks (Figure 2a). The amount of alcohol experienced by CIE by CIE-ED rats reached the desired range by the fourth week of vapor exposure as indicated by their BALs (Figure 2b). With respect to drinking sessions, a significant sessions × ethanol groups interaction (F(3,39) = 6.1, *p* = 0.001), main effect of session (F(3,39) = 6.4, *p* = 0.001) and main effect of group (F(1,13) = 6.4, *p* = 0.02) was obtained for ethanol consumed over the six weeks of vapor exposure. Further investigation of the interaction revealed that in CIE-ED rats, consumption of ethanol increased during weeks 4–7 compared to pre-vapor responding (*p*s < 0.05; Figure 2a). No difference in ethanol consumption was observed across weeks in ED rats. Also, from week 4 onwards, CIE-ED rats exhibited higher consumption of ethanol compared to ED rats (*p*s < 0.05).

### 2.2. CIE-ED Rats Demonstrate Somatic Withdrawal Symptoms

Withdrawal scores of body posture and tail stiffness were higher in CIE-ED rats compared with ED rats (*p*s < 0.05; Figure 4). Locomotor activity and grooming behavior were not different between the two groups.

### 2.3. ED and CIE-ED Rats Show Increased Number of CA II Immunoreactive Cells in the Hilus and 4-FS Reduces This Effect

**Figure 3:**
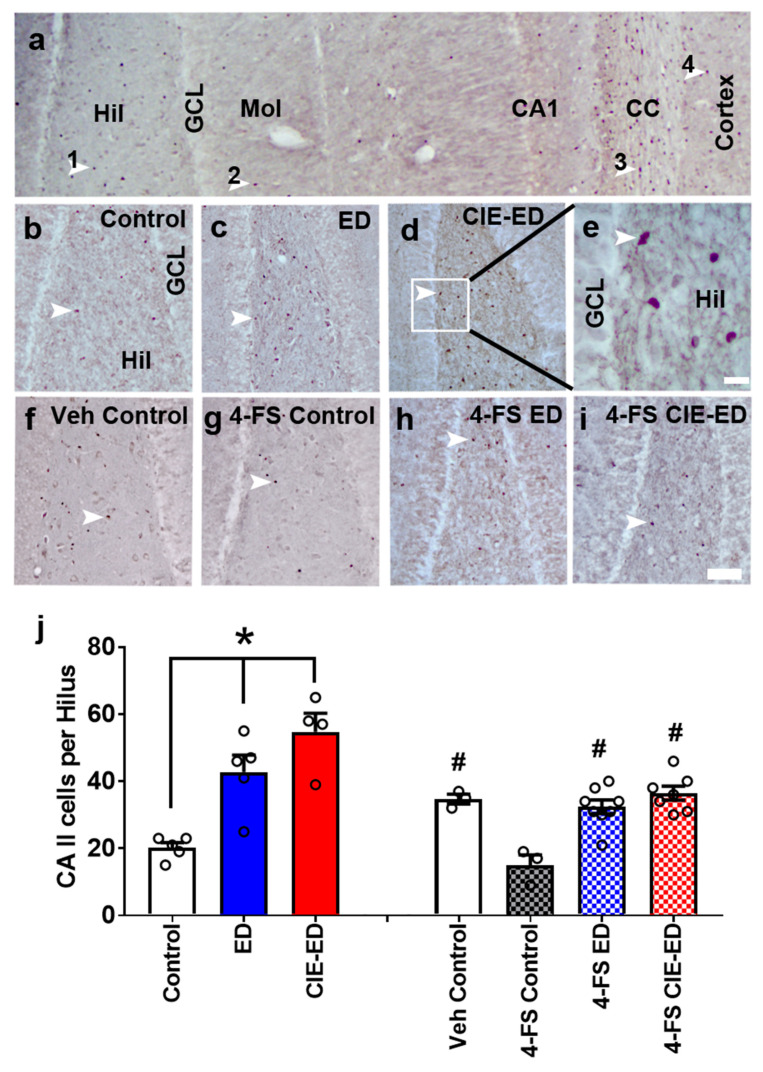
Carbonic anhydrase type II (CA II) expression in the adult rat hippocampus. (**a**) Photomicrograph of CA II immunohistochemistry in the hippocampus and cortex from one control rat. CA II+ cells appeared as single cells; each immunoreactive cell is pointed with an arrowhead. 1- CA II+ cell in the hilus (Hil); 2-CA II+ cell in the molecular layer (Mol); 3-CA II+ cell in the corpus callosum (cc); 4-CA II+ cell in the cortex. (**b**–**i**) 100× images of the hilus used for quantitative analyses of CA II cells. (**e**) Zoomed in image shown in (**d**) to indicate the morphology of CA II+ cells in the hilus. Scale bar in (**e**) is 20 µm; scale bar in (**i**) is 50 µm (applies **b**–**d** and **f**–**i**). (**j**) Number of CA II+ cells in the hilus. *n* = 5 controls, *n* = 5 ED, *n* = 4 CIE-ED, *n* = 3 vehicle controls, *n* = 3 4-FS controls, *n* = 8 4-FS ED rats, *n* = 7 4-FS CIE-ED rats. * *p* < 0.05, compared to controls; # *p* < 0.05 compared to 4-FS control. Data are expressed as mean ± S.E.M.

CA II immunoreactive cells were detected in the hilus and the corpus callosum regions (Figure 3a). Separate analyses were performed on controls, ED and CIE-ED rats that did not receive any 4-FS treatment. ED and CIE-ED rats that did not experience 4-FS treatment show increased number of CA II immunoreactive cells in the hilus compared to controls by one-way ANOVA (F(2,11) = 17.0, *p* = 0.0004). Post hoc analyses demonstrated higher number of CA II cells in ED and CIE-ED rats compared to their controls (*ps* < 0.01; Figure 3b–e,j).

4-FS treatment in ethanol-naïve rats reduced the number of CA II immunoreactive cells in the hilus and 4-FS treated CIE-ED and ED rats had the same number of CA II immunoreactive cells in the hilus compared with controls as shown by one-way ANOVA (F(3,17) = 11.9, *p* = 0.0002). Post hoc analyses demonstrated reduced number of CA II cells in 4-FS treated ethanol naïve rats compared with controls, 4-FS treated ED and CIE-ED rats (*p* < 0.01; Figure 3j).

### 2.4. 4-FS reduces Withdrawal Behavior in CIE-ED Rats and Reduces Drinking in ED Rats 

**Figure 4:**
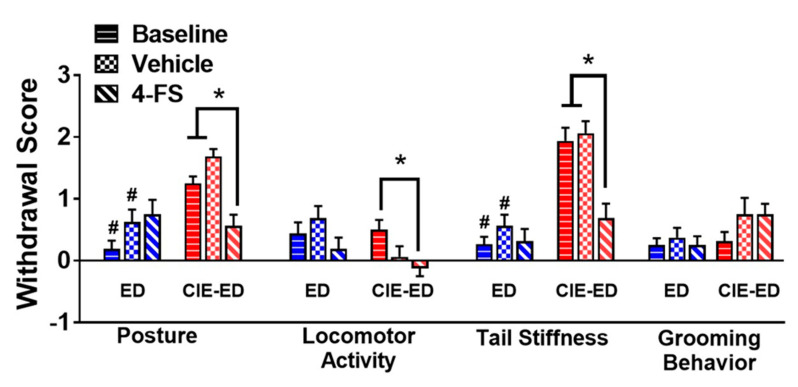
Physical withdrawal behaviors in ethanol drinking (ED) and CIE-ED rats with vehicle and 4-FS treatment. Withdrawal behaviors are evident in CIE-ED rats. Signs of ethanol withdrawal, i.e., aberrant body posture, locomotor activity and tail stiffness were reduced by 4-FS in CIE-ED rats. *n* = 7–8/group. * *p* < 0.05 compared to baseline and vehicle days, within-subject. # *p* < 0.05 vs. CIE-ED rats, between-subject. Data are expressed as mean ± S.E.M.

The effect of vehicle and 4-FS on physical withdrawal and drinking during withdrawal in CIE-ED and ED rats were determined as a within subject design during week 7 of ethanol sessions. 4-FS did not alter withdrawal scores in ED rats. 4-FS reduced withdrawal scores of posture and tail stiffness in CIE-ED rats bringing them to the levels of ED rats (*p*s < 0.05; Figure 4). 

**Figure 5:**
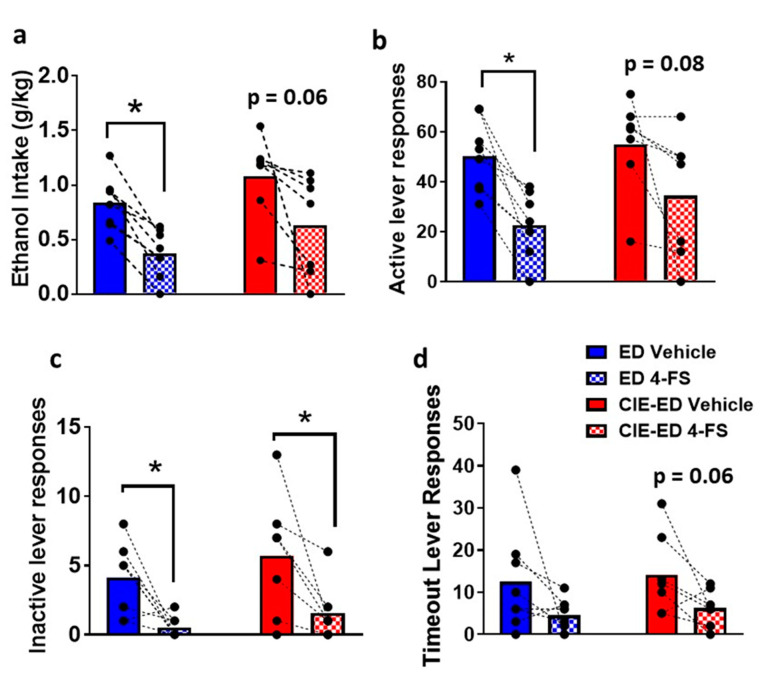
4-FS reduces drinking in ethanol drinking (ED) rats. (**a**) Ethanol intake expressed as g/kg and (**b**) active lever responses. (**c**) Inactive lever responses and (**d**) lever responses during timeout. *n* = 8 4-FS ED rats, *n* = 7 4-FS CIE-ED rats. * *p* < 0.05 vs. vehicle day, within-subject. Data are expressed as mean ± S.E.M.

The effect of 4-FS on ethanol self-administration in ED and CIE-ED rats were determined as a within subject effect (Figure 5). 4-FS reduced the amount of ethanol consumed and the associated active lever responses in ED rats (ethanol intake: *p* = 0.0004; active lever responses: *p* = 0.001; by paired t test Figure 5a,b). 4-FS did not significantly alter the amount of ethanol consumed or active lever responses in CIE-ED rats, however, showed a strong trend towards decrease compared with vehicle treatment (ethanol intake: *p* = 0.06; active lever responses: *p* = 0.08). 4-FS reduced inactive lever responses in CIE-ED and ED rats (CIE-ED: *p* = 0.04; ED: *p* = 0.01; Figure 5c). 4-FS did not alter lever responses during timeout in CIE-ED and ED rats (CIE-ED: *p* = 0.06; ED: *p* = 0.13; Figure 5d).

### 2.5. 4-FS Alters Expression of GluRs and GABA_A_Rs in the Ventral Hippocampus

Protein expression between vehicle controls and 4-FS treated controls did not differ and therefore they were combined as controls and used for analyses. 

**Figure 6:**
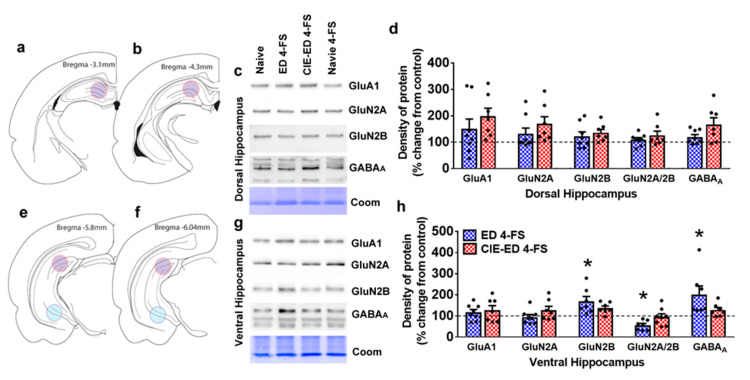
(**a**,**b**,**e**,**f**) Schematic of tissue punches from the dorsal hippocampus (**a**,**b**) and ventral hippocampus (**e**,**f**). (**c**,**g**) sample western blots from dorsal (**c**) and ventral (**g**) hippocampus tissue punches. (**d**,**h**) Densitometric analysis of tissue for levels of GluA1, GluN2A, GluN2B, ratio of GluN2A/2B and GABA_A_ from the dorsal (**d**) and ventral (**h**) hippocampus tissue homogenate. *n* = 8 4-FS ED rats, *n* = 7 4-FS CIE-ED rats. Values are mean ± S.E.M. expressed as % control, where control is represented by the dotted line in each graph. * *p* < 0.05, compared to controls.

In the dorsal hippocampus, there was a trend towards increase in GABA_A_ expression (one-way ANOVA GABA_A_: F(2,18) = 3.0; *p* = 0.07; Figure 6d). GluA1, GluN2A, GluN2B and the ratio of GluN2A/2B were unaltered (Figure 6d).

In the ventral hippocampus, GluN2B and GABA_A_ expression were increased (one-way ANOVA, GluN2B: F(2,18) = 1.5; *p* = 0.03; GABA_A_: F(2,17) = 1.6; *p* = 0.04). Post hoc analysis indicated higher expression of GluN2B in 4-FS treated ED rats compared with controls (*p* = 0.02), and higher expression of GABA_A_ in 4-FS treated ED rats compared with controls (*p* = 0.05). The ratio of GluN2A/2B expression was reduced (one-way ANOVA GluN2A/2B: F(2,18) = 1.0; *p* = 0.03; Figure 6h). Post hoc analysis did not detect any group differences in GluN2A/2B expression. 

### 2.6. 4-FS Alters the Number of Fos Cells in the Ventral Hippocampus

**Figure 7:**
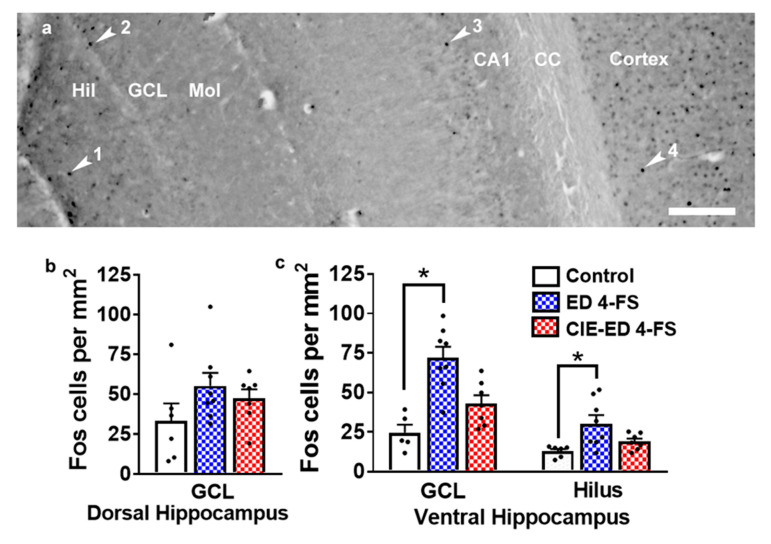
Fos expression is altered by 4-FS. (**a**) Photomicrograph of Fos immunohistochemistry in the hippocampus and cortex from one control rat. Fos+ cells appeared as single cells; each immunoreactive cell is pointed with an arrowhead. 1-Fos+ cell in the hilus (Hil); 2-Fos+ cell in the granule cell layer (GCL); 3-Fos+ cell in the CA1 region; 4-Fos+ cell in the cortex. Mol, molecular layer. Scale bar is 100 µm. (**b**,**c**) Number of Fos+ cells in the dorsal (**b**) and ventral (**c**) hippocampus. *n* = 8 4-FS ED rats, *n* = 7 4-FS CIE-ED rats. * *p* < 0.05, compared to controls. Data are expressed as mean ± S.E.M.

The number of Fos cells did not differ between vehicle controls and 4-FS treated controls and therefore, they were combined as controls and used for analyses. The number of Fos cells did not differ in the dorsal hippocampus in the granule cell layer (GCL); (F(2,18) = 1.7; *p* = 0.19; Figure 7b). In the ventral hippocampus, the number of Fos cells increased in the GCL and hilus regions (one-way ANOVA, GCL: F(2,18) = 12.2; *p* = 0.0004; hilus: F(2,18) = 5.9; *p* = 0.009; Figure 7c). Post hoc analyses indicate higher number of Fos cells in the GCL in 4-FS treated ED rats compared to controls (*p* = 0.0007) and 4-FS treated CIE-ED rats (*p* = 0.004) (Figure 7c). Post hoc analyses indicate higher number of Fos cells in the hilus in 4-FS treated ED rats compared to controls (*p* = 0.01) and 4-FS treated CIE-ED rats (*p* = 0.04) (Figure 7c).

## 3. Discussion

Our study demonstrates the expression of CA II in the adult rat hippocampus and corpus callosum, and that systemic 4-FS treatment reduces CA II expression in the hippocampus. In this study we also demonstrate a novel role of CA II in ethanol self-administration associated with non-dependent drinking and somatic withdrawal symptoms associated with ethanol dependence. For example, 4-FS reduced drinking in ED rats. 4-FS also demonstrated a strong trend towards reduction in drinking in CIE-ED rats, although this effect did not reach statistical significance. These findings imply that the dose of 4-FS required for reducing drinking in ED vs. CIE-ED rats may be different, and additional studies are required to address this issue. A more notable finding is that 4-FS reduced physical withdrawal behavior in CIE-ED rats without producing any non-specific effects in the ED rats. Postmortem tissue analyses revealed significant neurobiological changes in the hippocampus. Specifically in the ventral hippocampus, we show that 4-FS enhanced GABA_A_ receptor expression and Fos expression, and that these changes were associated with reduced ethanol consumption in ED rats. These findings highlight the importance of studying the role of CAs in AUD and may offer a potential new therapeutic approach to treat AUD.

The role of CAs in CNS disorders are fairly unexplored and needs investigation [44,45,46]. This is because physiological studies have demonstrated the expression and activity of CAs in the rodent and human CNS, particularly in the hippocampus [34,35,36,46,47]. Notably, the expression of CAs is abundant in oligodendroglia [48,49], and our immunohistochemical findings in adult rat tissue indicate CA II immunoreactivity in the hilus and molecular layer of the dentate gyrus and the corpus callosum, areas rich in oligodendrocytes. In addition, the lack of CA II expression in the granule cell layer and CA1 and CA3 pyramidal layers of the hippocampus supports the oligodendroglial expression of the protein. Given the abundant non-neuronal expression of CA II in the hippocampus, it is interesting to note that extracellular CA II can regulate neuronal transmission in the hippocampus [36,47,50]. For example, activity of CAs, specifically, CA II enables the rapid, reversible hydration of carbon dioxide, replenishing H^+^ ions, limiting interstitial alkalosis by preventing amplification of GluN and GABA_A_ receptor-mediated, bicarbonate-dependent alkaline shifts [34,51,52]. This is important because, neural activity, particularly GluN and GABA_A_ receptor-mediated activity in the hippocampus is accompanied by shifts in extracellular pH, which can influence normal and pathological brain function [53,54,55].

In the context of these studies, ethanol experience in rodents produces respiratory acidosis and metabolic acidosis that is accompanied by respiratory depression [56]. For example, it is likely that chronic ethanol experience will lead to brain tissue acidification and carbon dioxide retention [56,57]. These physiological changes by ethanol may induce increased tissue CA activity to maintain acid-base balance by rapid catalytic hydration of carbon dioxide to carbonic acid [58]. Additionally, it is possible that ethanol in vivo induces glutamate and GABA evoked alkaline shifts in the hippocampus, such that glutamate and GABA-induced efflux of bicarbonate from cells increases CA expression and activity to maintain generation of H^+^, and production of carbonic acid [59,60]. Acute withdrawal following high levels of intoxication produces respiratory alkalosis and hyperventilation, probably due to reduced levels of carbon dioxide – a physiological response that is observed in rodents and humans [57,61,62]. Interestingly, hyperventilation is associated with enhanced CA function [58,63]. These studies suggest that the acid-base imbalance in the brain in ED and CIE-ED animals are probably significant, and that the accompanying pathophysiological changes may induce aberrant levels of CA in the brain, particularly the hippocampus. Our results supports this hypothesis. 

The enhanced expression of CA in the hippocampus could modulate GluN and GABA receptor function in ED and CIE-ED rats [64,65]. For example, evidence from in vitro and in vivo studies suggests that glutamatergic and GABAergic neurotransmission are critical mediators of synaptic plasticity that may underlie alcohol dependence [19,64]. Particularly interesting is the increases in GluN receptor subunit expression and downregulation of GABA receptors in the hippocampus during CIE [14,18,64] and continued loss of GABA_A_ receptors and alterations in GABA_A_ receptor composition during withdrawal, which parallels the increased GluN subunit and reduced GABA_A_ expression in the hippocampus during epileptogenesis [64,66,67]. Although the neuroplasticity (imbalance of GluN and GABA) in the hippocampus may not directly regulate excessive drinking associated with dependence, the withdrawal symptomatology manifested as somatic symptoms could be driven by the hyperglutamatergic state in the hippocampus [68]. Moreover, the alterations in the hippocampus may be driven by the hyperglutamatergic state in the basolateral amygdala and other limbic regions which play a direct role in excessive drinking during dependence [69,70,71,72]. Taken together, the hyperactivity in the hippocampus stemming from the extended amygdala and the other limbic regions may be decisive factors for the maintenance of dependence in the long term [66,67,69,73].

As discussed above, the enhanced CA activity in the hippocampus in CIE-ED and ED rats may be occurring as a rebound effect in response to ethanol-induced acid-base imbalance in the hippocampus. Inhibitors of CAs are antiepileptic and reduce hyperexcitability induced by altered GluN and GABAergic signaling in the hippocampus [37,50]. Therefore, we used 4-FS, a novel and selective CA II inhibitor to determine whether inhibition of CA II would coincide with reduced drinking and physical withdrawal behavior in ED and CIE-ED rats. Our results show that 4-FS produced beneficial effects in ED and CIE-ED rats, indicating that this novel compound can be used to treat symptoms associated with moderate to severe AUD. Our findings are the first to demonstrate the protective effects of a CA II inhibitor in drinking behaviors in an animal model of moderate AUD and physical withdrawal behaviors in an animal model of severe AUD. A potential limitation in the interpretation of our findings is that only one type of CA II inhibitor (a sulfonamide) was tested in our study, and testing additional classes of CA II inhibitors may offer complete support to mechanisms underlying the protective effects of such inhibitors. However, we believe that these studies are beyond the scope of the current study. Nevertheless, our findings support other preclinical studies conducted with other sulfonamide anticonvulsants and nonselective CA II inhibitors, which have indicated protective effects of this drug class in ethanol drinking behaviors [74,75]. Our findings also support several clinical studies conducted with sulfonamides and their therapeutic effects in reducing risky drinking in alcohol dependent individuals [76,77,78,79]. Additionally, they support a previous publication performed in *ex vivo* hippocampal slice cultures, where a nonselective inhibitor of CA prevented binge ethanol-induced edema and neurodegeneration [80]. These findings suggest that CA II inhibitors are a promising therapeutic target to treat cellular toxicity and behavioral deficits associated with moderate to severe AUD.

We next attempted to determine the mechanism underlying the behavioral effects mediated by 4-FS. For example, pharmacological targets of clinically used CA inhibitors include the GABA_A_ receptors, glutamatergic receptors, sodium channels, potassium channels and calcium channels [81]. Notably, sulfonamides exert their effects by blocking sodium channels, reducing glutamate neurotransmission via direct actions on excitatory amino acid transporters and potassium channels and indirectly by enhancing GABAergic neurotransmission [77,82,83,84,85]. We therefore determined whether 4-FS treatment altered the expression of GABA_A_ and glutamatergic receptors in the hippocampus in ED and CIE-ED rats, due to the contribution of these receptor systems in the development and maintenance of alcohol dependence [19,64]. Furthermore we evaluated the expression of proteins along the dorsal/ventral gradient of the hippocampus. This is because, neuroanatomical studies in the hippocampus support segregation of neuronal outputs along the dorso-ventral axis whose connectivity may influence the expression of behavior dependent on the hippocampus [86,87]. The dorsal hippocampus is more plastic and is vital for spatial learning, and is particularly critical in mediating contextual discrimination [88]. However, the ventral hippocampus is less plastic and is strongly associated with negative affective symptoms, including craving, drug-seeking and increased consumption [89,90]. Similar functional differences along the septo-temporal axis of the hippocampus have been noted in humans, with ventral hippocampus demonstrating greater activity in response to negative affective symptoms [91]. Our findings demonstrate 4-FS-induced higher GABA_A_ expression, higher GluN2B expression and lower ratio of GluN2A to 2B in the ventral hippocampus in ED rats compared with control conditions, and these changes coincided with reduced drinking in ED rats. Therefore, enhanced GABA_A_ expression in the ventral hippocampus could have significant implications on GABAergic transmission and its role in regulating reduced drinking in 4-FS treated ED rats [64]. In addition, our results demonstrate altered expression of the GluN2B subunits and altered ratio of 2A to 2B in the ventral hippocampus in 4-FS treated ED rats. Altered expression of GluN2A been linked to impaired hippocampal-sensitive cognitive and electrophysiological function [92], and chronic ethanol experience alters expression of hippocampal GluN2A and GluN2B, findings which were attributed to the increases in the ratio of 2A/2B [18,93,94]. Therefore, reduced 2A/2B ratio in the ventral hippocampus could have significant implications on glutamatergic transmission and its role in regulating reduced drinking in 4-FS treated ED rats [95]. 4-FS did not produce any significant changes in the levels of GABA_A_ and GluNs in the hippocampus in CIE-ED rats. Therefore reduced physical withdrawal behavior observed in 4-FS treated CIE-ED rats could be occurring via mechanisms in the extended amygdala region, and investigating these mechanisms would be an important future pursuit [19].

We next investigated alterations in the expression of Fos, a marker for neuronal activity. The increases in GABA_A_ expression in ED rats in the ventral hippocampus paralleled the increases in the number of Fos immunoreactive cells in the GCL and hilus in the ventral hippocampus. Given that GABA_A_ receptor expression in somatostatin and parvalbumin neurons in the granule cell layer and hilus of the dentate gyrus controls inhibitory network activity in the hippocampus [96,97], it is possible that enhanced Fos activity occurred in either of the neuronal populations. Taken together, our findings demonstrate a new mechanism associated with ethanol consumption in nondependent subjects and physical withdrawal associated with ethanol dependence. Given that certain classes of CA inhibitors have been used for over six decades to treat various central and peripheral disorders, safely, and with minimal side effects [81], it is tempting to speculate that CA II inhibitors such as 4-FS will be a promising therapeutic approach to treat negative affective symptoms associated with moderate to severe AUDs.

## 4. Materials and Methods

### 4.1. Animals

Twenty-two adult male Long Evans rats (Charles River, Wilmington, MA, USA) were 8 weeks old at the beginning of the study, and weighed approximately 220–250 g. The rats were housed in reverse 12 h light-12 h dark cycle rooms and two/cage. Food and water were available ad libitum. All experimental procedures were approved by the Institutional Animal Care and Use Committee (protocol #A16-000, approved on 29 April 2016) at VA San Diego Healthcare System.

### 4.2. Ethanol Self-Administration

The behavioral experiments conducted herein are presented as a detailed schematic in Figure 1a. Sixteen experimentally-naive rats were given two 14-h lever-responding training sessions in the operant conditioning boxes (Med Associates Inc, St. Albans, VT, USA), on an fixed-ratio 1 schedule (FR1) for water followed by 30 min sessions of ethanol (10% *v*/*v*) that lasted for 10 days. These self-administration sessions of ethanol (training and maintenance) was performed according to our previous publication [20]. Following training and maintenance of ethanol self-administration, the rats were divided into two groups; one group experienced chronic intermittent ethanol vapor exposure (CIE; details of this procedure are available in our previous publication [20]) while the other group was exposed to air in their normal housing condition (did not experience ethanol vapors) for a duration of 6–7 weeks. Self-administration of ethanol continued during the 6–7 weeks of vapor or air exposure; all rats received two 30-min FR1 sessions per week (Tuesdays and Thursdays) during these 6–7 weeks. Based on these experimental conditions, these rats belonged to CIE-ED (*n* = 8) or ED (*n* = 8) groups, respectively. Lever responses were analyzed to determine escalation of self-administration compared to pre-vapor stable responding.

### 4.3. Tail Bleeding for Determination of Blood Alcohol Levels (BAL)

To measure and ensure consistent BALs, tail bleeding was performed on the CIE-ED rats, once a week (every Wednesday), between hours 13–14 of vapor exposure as detailed in our previous publication [20]. When plasma samples were outside the target range (125–250 mg/dL), vapor levels were adjusted accordingly. One CIE-ED rat was excluded from the study as the rat did not meet the criteria for ethanol dependence.

### 4.4. 4-FS Synthesis and Treatment

4-FS an inhibitor of CA type II was synthesized via a green synthetic method as reported in a recent publication [43]. 4-FS was dissolved in 6% DMSO in sterile water. 4-FS or equal volume of vehicle was injected at a dose of 20 mg/kg i.p., 20 min before physical withdrawal scoring. Few ethanol naïve rats (*n* = 3) received 4-FS 1 h before euthanasia.

### 4.5. Scoring of Physical Withdrawal

During the 7th week, for CIE-ED rats (or time matched for ED rats), physical withdrawal was evaluated for 1 min at 7 h post vapor cessation. Physical withdrawal measures were conducted on three separate days: day one—without any intraperitoneal injections (baseline), day two—20 min after vehicle injection, day three—20 min after 4-FS injection. This optimal time point was chosen for measuring physical withdrawal based on previous publications [98,99,100]. The 7 h post-vapor time-point corresponded with the peak of withdrawal following the termination of vapor exposure [20]. Withdrawal signs in rats were scored by two observers blinded to the dependence status of the rats using previously published rubric [68,101], and details on individual withdrawal signs of abnormal body posture, locomotion, tail stiffness and grooming movements were scored separately on a scale of 0 to 3 based on detailed description presented in our previous publication [20]. Although the behaviors scored could reflect seizure-like activity, seizures were not measured and were not scored.

### 4.6. Brain Tissue Collection

Forty-five min after the last drinking session, CIE-ED, ED and time and age-matched ethanol naïve rats were killed by rapid decapitation and the brains were isolated, and dissected along the midsagittal plane. The left hemisphere was snap frozen for Western blotting analysis and the right hemisphere was postfixed in 4% paraformaldehyde for immunohistochemistry as indicated in our previous publication [102].

### 4.7. Western Blotting

Western blot procedures were performed according to our previous publication [20]. Tissue punches from 2–3 500-µm thick sections containing the dorsal and ventral hippocampus sections (Figure 5) were homogenized by sonication in ice-cold buffer HEPES buffer, and protein concentration was determined using a detergent-compatible Lowry method (Bio-Rad, Hercules, CA, USA). 20 µg protein samples were subjected to gel electrophoresis and transferred to PVDF membranes.

The membranes were incubated with the following primary antibodies: total AMPA Receptor 1 antibody (GluA1; rabbit monoclonal; 1:500, cell signaling technology, Danvers, MA, USA, cat#13185, molecular weight 100 kDa), total glutamate (NMDA) receptor subunit 2A antibody (GluN2A; rabbit polyclonal, 1:200, Santa Cruz Biotechnology Dallas, TX, USA, cat# sc-9056, molecular weight 170 kDa), total glutamate (NMDA) receptor subunit 2B antibody (GluN2B; 1:200, Santa Cruz Biotechnology, Dallas, TX, USA, cat. no. sc-9057, molecular weight 180 kDa) and total GABA_A_ receptor antibody, PhosphoSolutions, Aurora, CO, USA, cat. no. 850-GA6, molecular weight 60 kDa). Following secondary antibody incubation, membranes were processed for immunoreactivity detection using SuperSignalWest Dura chemiluminescence detection reagent (Thermo Scientific, Waltham, MA, USA) and images were collected using a digital imaging system (Azure Imager c600, VWR, Radnor, PA, USA). For normalization purposes, membranes were incubated with 0.125% coomassie stain (cat # 1610400, Bio-Rad, Hercules, CA, USA) for 5 min and washed three times for 5–10 min in de-stain solution [103,104]. Densitometry was performed using ImageJ software (NIH, USA). The signal value of the band of interest was then expressed as a ratio of the corresponding coomassie signal. This ratio of expression for total protein was then expressed as a percent of the control sample included on the same blot.

### 4.8. Immunohistochemistry and Quantitative Analysis of Carbonic Anhydrase II (CA II) and Fos Labeled Cells

Tissue was sliced in 40 µm sections along the coronal plane in a cryostat. Two sections through the hippocampus were mounted on Superfrost^®^ Plus slides (Fisher Scientific, Hampton, NH, USA) and dried overnight and processed for CA II and Fos analysis. The following primary antibody was used for CA II immunohistochemistry (IHC): rabbit polyclonal, 1:500, catalog # PA5-78897, Invitrogen, Carlsbad, CA, USA) and for Fos IHC: (mouse monoclonal, 1:1000, catalog # sc-52, Santa Cruz Biotechnology, Dallas, TX, USA [105]). The sections were pretreated [106], blocked, and incubated with the primary antibody followed by biotin-tagged secondary antibody. Staining was visualized with 3,3′-diaminobenzidine chromogen (DAB; cat# SK-4100; Vector Laboratories, Burlingame, CA, USA).

For CA II analyses we used hippocampal tissue from control (*n* = 5), ED (*n* = 5) and CIE-ED (*n* = 4) rats that did not receive any 4-FS treatment. Behavior and other immunohistochemical data from these animals have been published elsewhere [17]. These animals were age matched compared to the animals used in the current study and consumed similar amount of ethanol via self-administration when compared to the animals used in the current study. We used the tissue from these animals to determine whether ED and CIE-ED enhanced CA II expression in the hippocampus. We also used tissue from the current study (vehicle controls, 4-FS controls, 4-FS ED, 4-FS CIE-ED) to determine whether 4-FS treatment altered CA II expression in the hippocampus. CA II immunoreactive cells in the hilus region of the dentate gyrus were examined and captured at 100× magnification (Figure 3b–i) with an AxioImager Microscope (Zeiss, Oberkochen, Germany). Cells in the hilus were visually quantified using ImageJ software and used for analyses.

For Fos analyses only tissue from the animals used in the current study were used. Fos immunoreactive cells were examined and quantified with a Zeiss AxioImager Microscope. Cell quantification was performed according to the methods described in our previous publication [107]. Fos immunoreactive cells were quantified in the dorsal hippocampus (dorsal granule cell layer (GCL) and ventral hippocampus (hilus and ventral GCL; Figure 7).

### 4.9. Statistical Analyses

Ethanol self-administration during the 7-week CIE exposure was evaluated using repeated measures two-way ANOVA with self-administration session as a within-subject factor and CIE as a between-subject factor. Significant interactions were investigated using Sidak’s post-hoc tests. Ethanol self-administration before and after 4-FS injection was compared using paired t-tests and self-administration between the CIE-ED and ED rats were compared using unpaired t-tests. Withdrawal scores at 7 h post-vapor in CIE-ED rats and time matched for ED rats were compared using a non-parametric Kruskal–Wallis one-way analysis of variance. For CA II, western blotting and Fos analyses, comparisons between control, CIE-ED and ED groups were made using one-way ANOVAs, followed by Tukey’s post-hoc tests. Statistical significance was set at *p* < 0.05.
